# Supplementary material for: Content-rich biological network constructed by mining PubMed abstracts
Source: BMC Bioinformatics. 2004 Oct 8;5:147. doi: 10.1186/1471-2105-5-147 (PMC528731; doi:10.1186/1471-2105-5-147)
Supplement: Additional File 5 — The original Chilibot query results of the term "long-term potentiation (LTP)" and 22 other terms, limiting the latest references analyzed to the years 1990, 1995, 2000, and 2004. [file 1471-2105-5-147-S5.bz2 › chilibotAdditionalFile5/ltp2004/html/SYNAPSIN I_TRKA.html]

 


 **SYNAPSIN I** and **TRKA** 
  
Found 1 abstracts in PubMed,  **1 abstracts were retrieved and analyzed**.  


---

 Search Google  |
 PDF files only 
|  EDU domain only 

---

- Neuropharmacology, 2003   **Cellular mechanisms of the acute increase of glutamate release induced by nerve growth factor in rat cerebral cortex.** .
  Nerve growth factor NGF was found to increase glutamate release in the developing visual cortex.
  We investigated the cellular mechanisms of this effect and its dependence on extracellular and intracellular Ca2ion .
  The NGF induced enhancement of glutamate release from superfused rat visual cortex synaptosomes required mild depolarization.
  Removal of external Ca2ion during depolarization with 15 mM K only halved the effect of NGF on glutamate release.
  NGF increased Ca2ion i in K depolarized synaptosomes preloaded with fura 2AM both in the presence and in the absence of external Ca2ion .
  The effects of NGF on glutamate release and Ca2ion i elevation were prevented by an anti **TrkA** receptor monoclonal antibody.
  NGF increased synaptosomal inositol ,5 triphosphate InsP3 during depolarization and the InsP3 receptor antagonist heparin abolished the effect of NGF on evoked glutamate release both in the presence and in the absence of external Ca2ion .
  The effect of NGF on the evoked glutamate release in Ca2ion free medium was abolished by dantrolene, a ryanodine receptor blocker, by CGP 37157, a blocker of the mitochondrial Na Ca2ion exchanger and by pretreatment of synaptosomes with caffeine.
  NGF significantly increased the depolarization induced activation of Ca2ion calmodulin dependent protein kinase II CaMKII and the subsequent phosphorylation of **synapsin I** in the absence of external Ca2ion and the NGF effect on evoked glutamate release was inhibited by the CaMKII inhibitors KN 93 and CaMKII 281 309 peptide but not by the MAP kinase inhibitor PD 98059.
  Thus, the effect of NGF on evoked glutamate release is linked to an increase in Ca2ion i contributed by both Ca2ion entry and mobilization from InsP3 sensitive, ryanodine sensitive and mitochondrial stores and to the subsequent activation of CaMKII.
